# Patient-derived tumor organoids for personalized cancer immunotherapy: An immunopeptidome-to-validation approach in RCC and BC

**DOI:** 10.1016/j.omton.2026.201247

**Published:** 2026-05-29

**Authors:** Gabriella Antignani, Michaela Feodoroff, Jacopo Chiaro, Sara Feola, Salvatore Russo, Firas Hamdan Hissaoui, Manlio Fusciello, Federica D’Alessio, Paolo Bottega, Yvonne Giannoula, Milda Sakalauskaite, Janita Sandberg, Miska Kosonen, Virpi Stigzelius, Tamara J. Luck, Valentina Ferrari, Daniele Ciampi, Markus Haapala, Rui Mamede Branca, Jukka Partanen, Satu Koskela, Maria Rescigno, Tiina Sikanen, Janne Lehtiö, Joseph Ndika, Otto K. Kari, Vilja M. Pietiäinen, Mikaela Grönholm, Vincenzo Cerullo

**Affiliations:** 1 Laboratory of Immunovirotherapy (IVT), Division of Pharmaceutical Biosciences, Faculty of Pharmacy, University of Helsinki, Helsinki, Finland; 2 Drug Research Program (DRP), Faculty of Pharmacy, University of Helsinki, Helsinki, Finland; 3 Translational Immunology Research Program (TRIMM), University of Helsinki, Helsinki, Finland; 4 Digital Precision Cancer Medicine Flagship (iCAN), University of Helsinki, Helsinki, Finland; 5 Helsinki Institute of Life Science (HiLIFE), University of Helsinki, Helsinki, Finland; 6 Institute for Molecular Medicine Finland (FIMM), Helsinki Institute for Life Sciences (HiLIFE), University of Helsinki, Helsinki, Finland; 7 Department of Biomedical Sciences, Humanitas University, Pieve Emanuele, MI, Italy; 8 Drug Research Program (DRP), Division of Pharmaceutical Chemistry and Technology, Faculty of Pharmacy, University of Helsinki, Helsinki, Finland; 9 Science for Life Laboratory, Department of Oncology-Pathology, Karolinska Institute, Solna, Sweden; 10 Finnish Red Cross Blood Service Biobank, Vantaa, Finland; 11 IRCCS Humanitas Research Hospital, Rozzano, MI, Italy; 12 VALO Therapeutics Ltd, Helsinki, Finland; 13 Department of Molecular Medicine and Medical Biotechnology and CEINGE, Naples University 24 Federico II, Naples, Italy

**Keywords:** personalized immunotherapy, PDTOs, immunopeptidomics, antigen discovery, HLA-peptides, CD8^+^ T cells, antigen-specific T cell response, cancer immunotherapy, renal cell carcinoma, bladder cancer

## Abstract

Immunotherapy has revolutionized cancer treatment, yet clinical success remains limited, with only a fraction of patients responding. Tumor heterogeneity and patient-specificity hinder response prediction, emphasizing the need for human-based models that accurately reproduce tumor-immune interactions. Conventional preclinical platforms, such as murine models, lack the human-specific HLA-TCR complexity, limiting their ability to accurately evaluate immunotherapy responses. To address this, we established a patient-specific pipeline for precision immunotherapy in renal cell carcinoma (RCC) and bladder cancer (BC). Tumor and adjacent tissues were used to generate patient-derived tumor organoids (PDTOs) and patient-derived cells (PDCs) for immunopeptidome profiling. Using our in-house microfluidic platform, PeptiCHIP, we identified tumor-associated HLA-I peptides as potential T cell targets. Their immunogenicity was evaluated using peptide-expanded, HLA-matched peripheral blood mononuclear cells (PBMCs), revealing peptides capable of inducing antigen-specific CD8^+^ T cell activation and cytotoxicity against the patient’s tumor. This study integrates PDCs, immunopeptidomics, and functional immune assays to design and test personalized cancer immunotherapies. By recreating patient-specific tumor-immune interactions *ex vivo*, our platform enables the discovery of therapeutic targets, the evaluation of immune responses, and validation in a patient-specific context. This approach demonstrates the feasibility of bridging a major gap in translational immunotherapy research and supports the development of personalized cancer immunotherapy strategies.

## Graphical abstract

**Figure:**
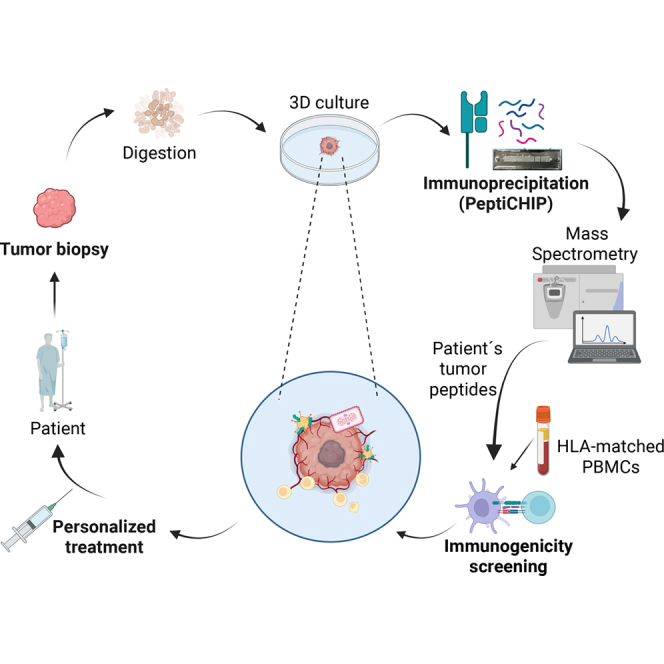

## Teaser

A patient-specific immunotherapy pipeline integrates tumor organoids, immunopeptidomics, and functional T cell assays to identify actionable tumor antigens. This approach enables *ex vivo* validation of personalized antigens and supports the development of individualized T cell-based cancer therapies.

## Introduction

Cancer immunotherapy has revolutionized tumor treatment approaches, yet its clinical implementation is often limited. Tumor heterogeneity and patient-specific differences are major contributors to treatment failure, with only a subset of patients benefiting from immunotherapies. Predicting responders in advance would greatly enhance clinical outcomes.

A prominent gap in the field remains the lack of reliable and representative human-based testing platforms to validate the efficiency of cancer immunotherapies. Currently, available preclinical models present several limitations. Murine models lack the human-specific tumor-immune interactions critical for evaluating immunotherapies reliant on HLA-TCR interactions.^1^ Humanized mice are more relevant but are costly, complex, and time-intensive.^2^ On the other hand, 2D cell cultures are practical and cost-effective but fail to capture the complexity of *in vivo* tumors, leading to poor translation of findings to clinical success.^3^

In the search for more predictive models, 3D cultures such as organoids have been introduced.^4^ Organoids are self-organized 3D structures that mimic *in vivo* tissues, with the first intestinal organoids developed by Hans Clevers’s group in 2009.^5^ This breakthrough enabled the establishment of patient-derived tumor organoids (PDTOs), which can be grown from primary patient material, expanded in a clinically meaningful time window, cryopreserved, and maintained long term.^2^ PDTOs closely reflect the genomic landscape and drug responses of the source tumor—showing correlation between drug screening *in vitro* and respective patient’s response to the same treatment^6^—and currently provide the closest *in vitro* system to recapitulate the original patient cancer tissue,^7^ making them powerful preclinical models for cancer research and precision medicine. A major challenge in immuno-oncology is replicating the complexity of the immune system behavior toward the tumor, but recent advancements in tumor-immune co-cultures have begun to reveal both opportunities and limitations of this emerging approach.^8^

Renal cell carcinoma (RCC) and bladder cancer (BC), two distinct cancers of the urinary tract, are highly immunogenic tumors, making them strong candidates for antigen-based immunotherapies.^9^^,^^10^ RCC, the most common kidney cancer, is typically treated with surgery for localized disease^11^ and demonstrating promising improvements in patient survivability when coupled with immunotherapy.^12^ Despite its low tumor mutational burden (TMB),^13^ the immune-rich microenvironment of RCC, including T cell infiltration and tumor-associated antigens like carbonic anhydrase (CAIX), supports its immunogenicity.^14^ Its pathogenesis is often driven by VHL (Von Hippel-Lindau) mutations, leading to angiogenesis and cell proliferation. In contrast, BC is linked to environmental factors such as smoking and chronic inflammation.^15^ It exhibits T cell infiltration and high immunogenicity at all stages, responding well to strategies like Bacillus Calmette–Guérin (BCG) therapy and checkpoint inhibitors.^16^ The high TMB of BC further enhances its responsiveness to immunotherapy, making it a valuable model for studying immune responses.^17^

This study aimed to explore a human-based, patient-specific pipeline to support precision immunotherapy for RCC and BC, as well as to design a tailored approach to the patient’s cancer. Tumor and adjacent benign tissues from RCC (*n* = 2) and BC (*n* = 3) patients were used to generate 3D PDTOs. For convenience, these 3D PDTOs were then plated on plastic or low-adhesion conditions and grown in 2D as patient-derived cells (PDCs) for further applications.

Tumor and matched benign tissues were obtained from surgical specimens and classified according to standard clinical pathology procedures. Tissue identity was determined based on macroscopic features, such as anatomical location, color, and texture, and, when necessary, confirmed by histological evaluation of stained sections. In this context, the benign tissue corresponds to the surrounding zone between malignant cells and normal tissue, representing an area that is not healthy yet less malignant than the tumor core. All RCC tumor samples included in this study correspond to clear cell renal cell carcinoma (ccRCC).

Tumor biopsies are challenging to work with as they offer small amounts of biological material, making peptide identification quite challenging. To investigate the immunopeptidome landscape, we used PeptiCHIP,^18^ an in-house microfluidic-based platform, to isolate antigens presented by MHC molecules. This advanced immunopeptidome technique requires minimal biological material compared to classical ligandome techniques, making it suitable for limited sample availability.

Identifying optimal tumor antigens remains a key challenge in T cell-based cancer immunotherapy. In this study, we present a patient-specific pipeline that integrates tumor-derived peptide identification with functional immune validation. Peptides obtained from PeptiCHIP were selected using a bioinformatics workflow^19^ and evaluated for immunogenic potential using HLA-matched healthy donor PBMCs. Antigen-specific CD8^+^ T cell responses were assessed in co-culture systems incorporating autologous antigen-presenting cells and patient-derived tumor cells. This approach combines tumor profiling, antigen selection, and immune functional testing to support the rational design of personalized cancer immunotherapies.

This work shows the feasibility of selecting relevant peptides using this pipeline, supporting its future expansion to larger patient cohorts. Further studies are needed to evaluate its applicability in preclinical immunotherapy research across RCC, BC, and other immunogenic cancers.

## Results

### Characterization of patient-derived samples: Tumor mutations and marker expression

**Figure 1:**
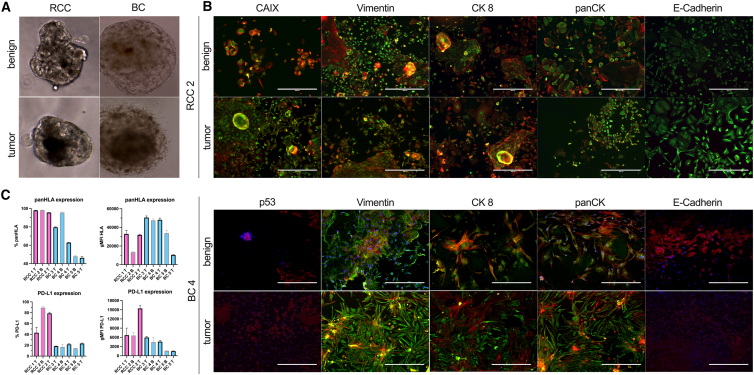
PDC characterization: morphology, marker expression, and population heterogeneity of PDCs (A) Bright-field microscopy images of PDTOs from RCC and BC cells cultured in 3D in 30% supportive Matrigel. The top row displays representative benign samples from RCC and BC, while the bottom row shows representative tumor samples from each. (B) Immunofluorescence staining to visually assess the heterogeneity of patient-derived cells (PDCs) in terms of cell cohorts. Benign and tumor RCC PDCs were stained for CAIX (green), vimentin (green), cytokeratin 8 (green), and pan-cytokeratin (green), all co-stained with phalloidin (red) to visualize the cytoskeleton; E-cadherin (red) was co-stained with CAIX (green). Benign and tumor BC cells were stained for vimentin (green), cytokeratin 8 (green), pan-cytokeratin (green), all co-stained with phalloidin (red) to visualize the cytoskeleton. In addition, BC PDCs were assessed for p53 (red) and E-cadherin (red) expression. In some of the pictures, DAPI (blue) is visible as well. One representative patient is shown for each cancer type. Scale bars, 400 μm. (C) Measurement of expression levels of surface markers HLA (top row) and PD-L1 (bottom row) in benign and tumor PDCs through flow cytometry. Percentage of expression and gMFI are shown for all patients.

PDTOs of two RCC patients (RCC1 and RCC2) and three BC patients (BC3, BC4, and BC5) were established from tumor and benign samples (benign tissue was available for RCC2, BC4, and BC5 only) obtained from surgical resections and were cultured in 3D on supportive Matrigel (Figures 1A and S1A) to better preserve their biological characteristics. Subsequently, 3D PDTOs were dissociated for cell counting and further expanded also in 2D culture, either on plastic or under low-adhesion conditions, to facilitate Matrigel removal from the cell suspension and increase PDC yield for downstream analysis. Characterization of 2D PDCs’ composition was done using cell population markers (Figure 1B) and quantification of HLA and PD-L1 expression levels (Figure 1C).

#### Immunofluorescence staining showed heterogeneous cell cohort which reflects the composition of the TME

To characterize cell populations in the cultured patient’s tissue biopsies, PDCs were seeded on glass slides, stained with different cell population markers, and analyzed for expression by fluorescence microscopy (Figures 1B and S1B). Populations showed high levels of heterogeneity, highlighted by distinct cell cohorts: tumor cells identified by upregulation of vimentin expression, CAIX (typically expressed in RCC), and p53 (often upregulated in response to stress in BC^20^), fibroblasts marked by vimentin, and epithelial cells characterized by cytokeratin and E-cadherin expression.^21^ Both benign and tumor PDCs seem to retain different components of the tumor microenvironment (TME) from the patient's biopsies, with tumor samples showing a clearer presence of tumor-associated markers compared to their benign counterparts (Figures 1B and S1B).

#### Fluorescence-activated cell sorting analysis revealed suitable HLA expression for downstream ligandome analysis

Prior to immunopeptidome analysis, we confirmed that our PDCs expressed sufficient HLA on the cell surface to ensure that retrieving peptides from the HLA complex would be feasible. Flow cytometry was used to evaluate the expression levels of HLA and PD-L1 surface markers in benign and tumor PDCs. The results showed high HLA expression, indicating that these patients are well-suited for ligandome analysis. In all patients, pan-HLA % was expressed on at least 40% of acquired cells compared to the unstained, ranging from 40% to 80% in BC (lowest in BC5) and close to 100% in RCC (Figure 1C). No major differences between benign and tumor samples were observed, except for BC4, which showed lower HLA levels in the tumor compared to the benign counterpart, and a slight pan-HLA increase for geometric mean fluorescence intensity (gMFI) in RCC2 tumor and a decrease in BC5 tumor compared to their benign samples.

As PD-L1 is a crucial protein for tumor immune escape, PD-L1 expression on tumor samples was checked to assess the potential for immune evasion via the PD-1/PD-L1 pathway. In contrast to pan-HLA, PD-L1 expression was relatively low (about 20%) in all BC samples and higher in RCC (from 40% to 80%), suggesting that PD-L1 is not strongly upregulated in these samples. As for gMFI, PD-L1 was found to be higher in RCC2 tumor compared to benign, while no visible differences between benign and tumor were observed in the rest of the patients (Figure 1C).

#### WES analysis of patient tissues highlighted tumor mutations

To further characterize our patient samples’ mutational profiles, whole-exome sequencing (WES) analysis of benign and tumor samples was used. Benign tissues exhibited lower TMB compared to their tumor counterparts, consistent with the higher mutational load characteristic of malignant and more advanced tumor stages^22^ (Figure S1C). Both RCC and BC patient samples harbored known somatic mutations.^23^^,^^24^ In particular, the RCC2 tumor displayed a notable TMB together with established driver mutations, such as *VHL*, while the BC5 tumor carried alterations in *APC* and *TP53* (Figure S1C). Coding non-synonymous somatic variants affecting exons and/or splice sites with a variant allele frequency ≥ 5% in the tissue were identified. Genes shown in the heatmap are those affected by variants predicted to be pathogenic or likely pathogenic by InterVar^25^ and/or considered functionally or clinically relevant by the personal cancer genome reporter (PCGR).^26^

#### Genomic comparison of tumor tissue and derived cultures revealed 3D culture outperforms 2D culture in retaining tumor mutations

Among the available samples, RCC2 provided sufficient material and robust growth, allowing its use as a representative model for deeper molecular characterization. Variant allele frequency (VAF) plots (Figure S1D) show the distribution of somatic mutations across the three RCC2 samples: tumor tissue (T), 3D culture derived from tumor tissue (T 3D_PDTOs), and 2D culture derived from tumor tissue (T 2D_PDCs). The *y* axis represents VAF, while the *x* axis shows the three sample types. Each colored dot represents a unique mutation, and identical variants detected in multiple samples are connected by lines. Points labeled with the same gene name but not connected represent distinct mutations within the same gene.

All three samples retained the *VHL* mutation, although with decreasing VAF values, consistent with reduced tumor fraction during *in vitro* expansion. Additional tissue mutations, including *CDX2* and *LEF1*, were preserved in 3D culture but were not detected in 2D. Some mutations present at low VAF in the fresh tissue were not maintained in either culture condition, suggesting loss of minor sub-clonal populations during model establishment. Both PDC culture models harbor mutations in m*TOR*; however, these mutations are distinct and therefore appear as unconnected points in the plots, indicating independent mutational events rather than shared variants.

Overall, three out of six variants detected in the tumor tissue were preserved in 3D culture, whereas only one out of six was detected in 2D. In addition, shared variants show higher VAF values in 3D than in 2D. Comparison of mutation profiles shows that the 3D culture retains a larger fraction of tissue-derived variants than the 2D culture.

### Immunopeptidome analysis and peptides selection pipeline allowed the identification of patient-specific tumor peptides

In total, 8 samples were analyzed: 2 tumor-only samples (RCC1 and BC3) and 6 paired benign and tumor samples (RCC2, BC4, and BC5). Each sample was run in duplicate, yielding 16 experimental samples.

#### Overview of *in vitro* and in silico pipeline for tumor-specific peptide identification and prioritization

**Figure 2:**
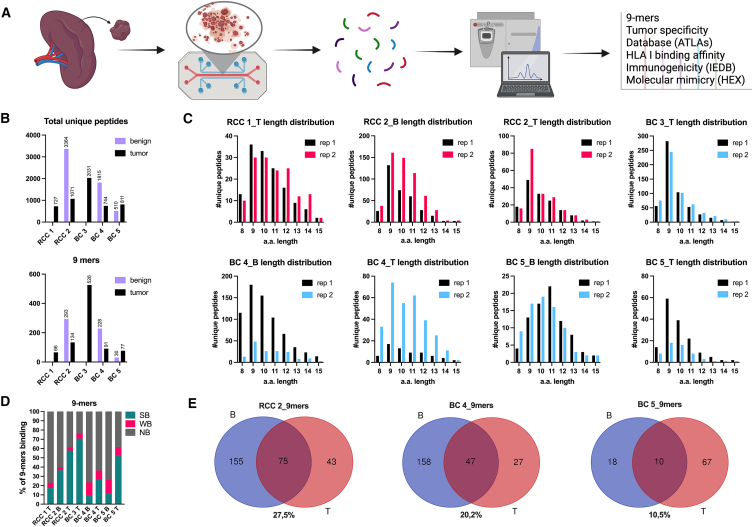
HLA immunopeptidome profile of patient-derived cells using PeptiCHIP (A) Schematic illustration of the criteria for the *in vitro* process and subsequent *in silico* peptide selection. First samples were lysed and run in PeptiCHIP, then eluted peptides were identified by mass spectrometry, and data were analyzed *in silico*. Data cleaning and curation were performed manually. (B) Eluted peptides count. Replicates (*n* = 2) for each sample were pooled together (benign cells were not available for patients RCC1 and BC3). The number of unique peptides across all lengths, as well as specifically for 9-mers, is displayed. (C) Distribution of eluted peptide lengths for each sample. An 8–15-mer-length interval is shown as an overview of quality control for eluted peptides. (D) Stacked bar plot depicting HLA binding affinity predictions. Percentages of 9-mers predicted to be strong binders (rank score < 0.5), weak binders (rank score < 2), or non-binders in each sample. The analysis was performed using the machine learning-based MHC-binding affinity tool MHCflurry, inserting the corresponding set of MHCs for each patient. (E) Venn diagrams showing the overlapping number and percentage of 9-mers between benign and tumor samples for each patient. Pools of 9-mers were considered, and replicates (*n* = 2) of the runs were pulled together for each sample.

To identify the most promising tumor-specific peptides, the *in vitro* sample processing and peptide isolation were followed by a stepwise *in silico* selection (Figure 2A).

To isolate peptides presented on HLA molecules from limited patient-derived material, we used PeptiCHIP (a microfluidic chip, which can capture class I complexes onto the pan-HLA antibody-coated solid surface) on PDCs. The eluted peptides were subsequently characterized by liquid chromatography-tandem mass spectrometry (LC-MS/MS) using a human canonical proteome as a reference database for spectral matching.

Prior to downstream in silico analyses, the resulting peptide lists were subjected to a manual clean-up step based on predefined physical criteria. This filtering step was implemented to reduce the technical noise of mass spectrometry detection by removing the contaminants and low-confidence identifications commonly observed in immunopeptidomics workflows.

We wanted to select peptides with the highest likelihood of being immunologically relevant, meaning those that could be both tumor-specific and capable of eliciting an immune response. To do so, we first prioritized 9-mer peptides, as this length is optimal for stable and effective binding to HLA class I molecules.^27^ Next, we filtered for peptides detected in tumor tissues but absent in their matched benign counterparts, ensuring that the selected targets were uniquely or preferentially expressed in malignant cells (as determined by Venn diagram-based separation). We then examined the source genes of these peptides using resources such as The Human Protein Atlas to confirm their expression profiles and tumor relevance. At this stage, we prioritized peptides with strong predicted HLA-binding affinity according to NetMHCpan 4.1, though we occasionally retained weak binders when other characteristics suggested potential immunogenicity. Additionally, to determine whether these peptides had been previously associated with T cell recognition, we queried the Immune Epitope Database (IEDB). As peptide binding affinity to HLA is not always a reliable predictor of immunogenicity, in our peptide selection pipeline, we tried to include additional aspects such as peptide “foreignness” or “non-selfness,” as proposed by Balachandran et al.,^28^ which may serve as a more accurate determinant of epitope immunogenicity. To achieve this, we used an in-house and publicly available software called Homology Evaluation of Xenopeptides (HEX),^29^ a modeling tool that exploits molecular mimicry to predict immunogenic sequences. This tool highlighted whether our peptides had similarity to known viral epitopes and helped us prioritize peptides most likely to trigger a measurable immune response in subsequent experiments.

#### QC of ligandome runs further validated the reliability of our peptide selection pipeline

As a quality control (QC) of our runs, we checked the amount of total unique peptides (duplicates of the same sequence were removed) as well as 9-mers. Across all samples, we observed a number of eluted unique peptides ranging between 500 and almost 3,000 per sample; and among these, 9-mers covered from 30 to 500, with BC3 resulting in the highest number of eluted 9-mers (Figure 2B).

Analysis of peptide length distributions revealed a clear enrichment for 9-mer peptides across samples, with a pronounced peak at nine amino acids (Figure 2C). This distribution is consistent with the expected characteristics of HLA class I ligandomes and aligns with the previously reported immunopeptidomics datasets,^30^ supporting the technical validity of the peptide isolation and identification workflow.

#### HLA-binding affinity prediction and tumor specificity of eluted peptides

To distinguish real HLA-I binders from potential contaminants, we employed machine learning-based algorithms for peptide-HLA binding prediction, assessing the binding affinity of our eluted peptides. NetMHC4.1 was applied to all identified 9-mer peptides in our dataset, revealing the percentages of 9-mers predicted to bind the respective HLA alleles for each patient (Figure 2D). BC3 T resulted in the sample with the higher % of binders (as expected, given the largest pool of eluted peptides), followed by RCC2 T and BC5 T. Percentage refers to the number of strong binders (rank score < 0.5), weak binders (rank score < 2), or non-binders in the pool of total 9-mers per each sample.

At this point, we wanted to see which peptides were found in tumor samples, but not in benign samples (when available), in order to extrapolate sequences that were specifically presented on the tumors. We used Venn diagrams to visualize the 9-mers overlap between benign and tumor samples of each patient (replicates pooled), and we found 43 tumor-specific 9-mers for RCC2, 27 tumor-specific 9-mers for BC4, and 67 tumor-specific 9-mers for BC5 (Figure 2E).

Additionally, we checked the number of shared peptides as well as shared 9-mers found in the two replicates of the same sample. The % of overlapping peptides among both replicates was in line with what is expected (Figure S2).

#### HLA binding motifs of eluted peptides

**Figure 3:**
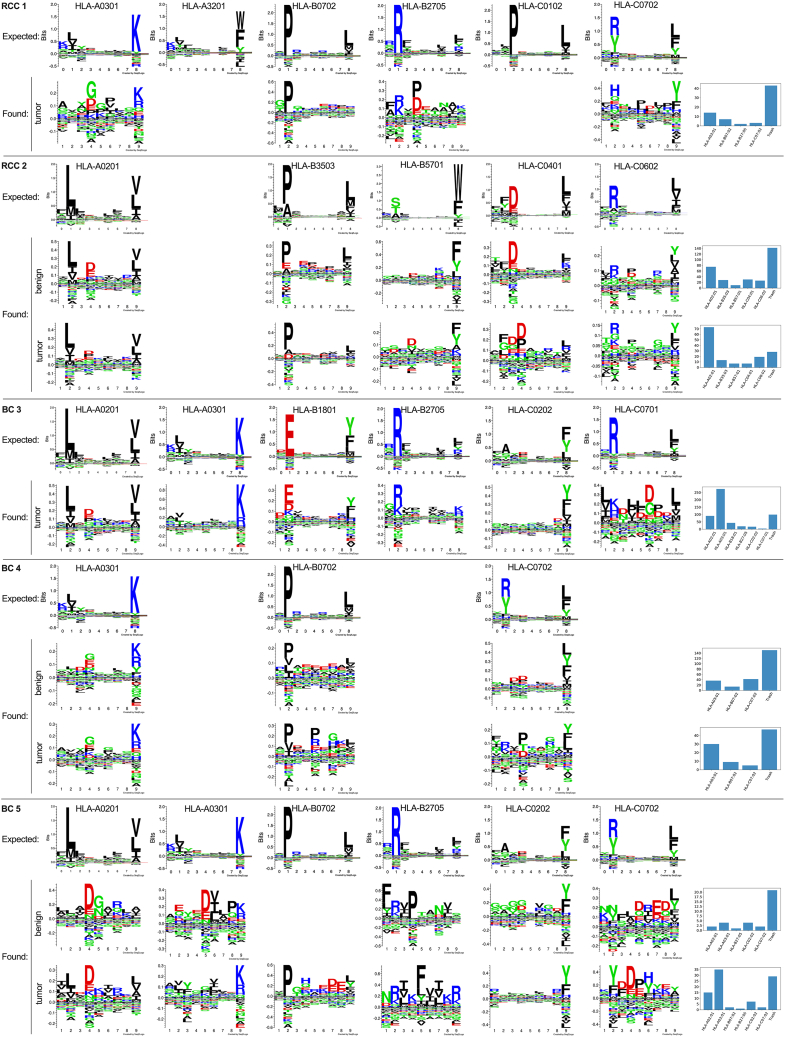
Eluted peptides HLA binding specificity deconvolution and HLA motifs comparison Comparison between the expected motifs of patients’ HLA alleles obtained from NetMHCpan/Motifs viewer (“expected”), and the results of the motif deconvolution method Gibbs clustering (“found”). The histograms represent the distribution of peptides assigned to the HLA alleles expressed by each patient. The *x* axis corresponds to the HLA alleles of each patient and the *y* axis corresponds to the number of peptides detected for each HLA allele. Bar plot shows peptide specificity deconvolution using machine learning predictions from MHCMotifDecon v1.0. Peptide specificity is annotated by using the best predicted HLA allele per peptide.

To further examine the quality of our ligandome datasets, we investigated whether the HLA-I binding motifs of the eluted peptides were matched with the ones expected (obtained from NetMHCpan/Motifs viewer). We have used MHCMotifDecon v1.0 as a supervised method that generates a motif after binding affinity deconvolution. We identified most of the expected binding motifs for all the HLA alleles; missing identification of an expected binding motif could depend on an insufficient number of peptides per allele or shared key anchor positions between two or more alleles. The bar plot displays the deconvolution of peptide specificity using machine learning predictions from MHCMotifDecon v1.0 where each peptide was assigned to its most likely presenting HLA allele (Figure 3).

In the end, we selected overall 52 patient-specific tumor peptides (Table 1). Additionally, we observed that some of our selected peptides (*n* = 11) or different peptides from the same source proteins (*n* = 17) were also found in a ligandome of the RCC cell line 786-O that we have previously analyzed.^31^

**Table 1:** Selected tumor peptides from 2 RCC and 3 BC patient samples after ligandome and bioinformatics analysis

Patient	MIX	ID	Peptide	Protein name	Allele	HD1	HD2	HD3	786-O	Functionality	Clinical relevance	Overexpression
RCC 1	MIX A	1	GVHGGLINK	PFN1	HLA-A03:01	x	x	x	p	actin cytoskeleton remodeling	prognostic and targetable	in RCC and BC
		2	AVYGGFKSK	ADH1B	HLA-A03:01	x	x	x	–	ethanol Metabolism	polymorphisms linked to cancer risk	–
		3	AAAKPVATK	COL6A3	HLA-A03:01	x	x	x	sp	ECM remodeling and stromal–tumor interactions	prognostic biomarker	in RCC and BC
		4	KTVDGPSGK	GAPDH	HLA-A03:01	x	x	x	sp	angiogenesis, metabolic reprogramming	prognostic biomarker	in RCC and BC
		5	KHSLPDLPY	SOD2	HLA-C07:02	x	x	x	–	oxidative-stress balance	tumor cell survival	in RCC
	MIX B	6	ATYGKPVHH	RPL15	HLA-A03:01	x	x	x	p	ribosomal protein, involved in protein translation	cancer cell proliferation	–
		7	VHERHETTY	FLG2	HLA-C07:02	x	x	x	–	skin barrier	–	–
		8	GVHGGILNK	PFN3	HLA-A03:01	x	x	x	p	cytoskeleton	–	–
		9	AENEFVTLK	KRT6A	HLA-A03:01	x	x	x	–	intermediate filament family	associated with aggressiveness and poor prognosis	in BC
RCC 2	MIX C	10	NLLPKLHIV	CLIC1	HLA-A02:01	x	x	x	–	ion-channel dynamics	associated with invasion and poor prognosis	in RCC and BC
		11	TRSVSSSSY	VIM	HLA-C06:02	x	x	x	sp	epithelial-mesenchymal transition	correlated with tumor stage and poor prognosis	in RCC and BC
		12	YLLPKDIKL	RASL11A	HLA-A02:01	x	x	x	–	cytoskeletal dynamics and cell motility	tumor suppressor	–
		13	SLFGGSVKL	PDCD6IP	HLA-A02:01	x	x	x	–	endosomal sorting, cytokinesis, and membrane trafficking	tumor progression and metastasis	in RCC and BC
		14	SLTGHISTV	PLRG1	HLA-A02:01	x	x	x	sp	spliceosome component; functions in pre-mRNA splicing	tumorigenesis	–
	MIX D	15	FLYDDNQRV	TOP2A	HLA-A02:01	x	x	x	sp	DNA topology and genomic stability	cancer cell proliferation	in RCC and BC
		16	YLDPAQRGV	METTL26	HLA-A02:01	x	x	x	–	methyltransferase	–	–
		17	TLDAGNIKL	PRPF40A	HLA-C04:01	x	x	x	sp	spliceosome assembly and RNA processing	–	–
		18	ALLGKIEKV	GALNT2	HLA-A02:01	x	x	x	–	O-linked glycosylation; linked to cell-cell interactions	tumor invasion and immune recognition	in BC
		19	LLDRFLATV	CCNI	HLA-A02:01	x	x	x	–	regulates cell cycle	cancer cell survival and proliferation	–
	MIX E	20	VLAPRVLRA	RCN1	HLA-A02:01	x	x	x	–	calcium-binding protein; involved in ER stress response	metastasis	–
		21	ALASHLIEA	EHD2	HLA-A02:01	x	x	x	sp	ATPase involved in membrane remodeling and endocytosis	tumor migration and invasion	in RCC
		22	LLDEEISRV	QKI	HLA-A02:01	x	x	x	sp	RNA-binding regulation	tumor suppressor	in RCC
		23	FASHVSPEV	ARFGAP3	HLA-A02:01	x	x	x	–	endocytosis	prognostic biomarker in RCC	–
		24	KIAPNTPQL	NOMO1	HLA-A02:01	x	x	x	–	modulator in nodal signaling	–	–
BC 3	MIX A	1	ALDQKVRSV	MAP1A	HLA-A02:01	x	x	–	–	immune cell infiltration	prognostic biomarker	–
		2	ALQKRLDEV	RRBP1	HLA-A02:01	x	x	–	sp	endoplasmic reticulum (ER) membrane protein	potential oncogene, associated with chemoresistance	in RCC and BC
		3	KVYGPGVAK	FLNA	HLA-A03:01	x	x	–	p	cell migration and cytoskeletal organization	tumor progression and metastasis	in RCC and BC
		4	GTAPAFKQK	MYLK	HLA-A03:01	x	x	–	p	cell contraction and motility	tumor invasion and metastasis	in RCC
		5	GTNKVASQK	CNN3	HLA-A03:01	x	x	–	–	cytoskeletal organization	tumor migration and invasion	in RCC
	MIX B	6	KVSTSPLTK	TMX2	HLA-A03:01	x	x	–	sp	redox regulation	tumor progression	–
		7	ATDPQTKRK	SEC31A	HLA-A03:01	x	x	–	–	part of the COPII complex, involved in protein transport	cancer cell proliferation	–
		8	IIYKGGTSR	GSN	HLA-A03:01	x	x	–	p	actin filament remodeling	cancer cell motility and metastasis	in BC
		9	RIDGPTGQK	LTBP1	HLA-A03:01	x	x	–	–	TGF-β signaling	cancer progression	in RCC
		10	SAYGSVKAY	ANXA2	HLA-C02:02	x	x	–	–	cell membrane repair	cancer cell invasion and metastasis	in RCC and BC
BC 4	MIX C	11	YTRPTPVQK	DDX3X	HLA-A03:01	–	x	x	p	RNA processing	cancer progression	in RCC
		12	ASSKDAIKK	CFL2	HLA-A03:01	–	x	x	–	cytoskeletal dynamics	cancer cell motility	in BC
		13	AVDSRTGKL	DSG1	HLA-B07:02	–	x	x	–	cell adhesion	cancer progression	–
		14	EVETPKNEL	DSC3	HLA-B07:02	–	x	x	–	cell adhesion	cancer progression	–
	MIX D	15	ASYGVSKGK	HNRNPU	HLA-A03:01	–	x	x	sp	RNA processing	cancer progression	–
		16	AVAIKAMAK	EIF5A	HLA-A03:01	–	x	x	p	protein synthesis	cancer progression	in RCC
		17	RIGKVGNQK	PSMD7	HLA-A03:01	–	x	x	p	protein degradation	cancer progression	in RCC
		18	YVTTSTRTY	VIM	HLA-C07:02	–	x	x	sp	epithelial–mesenchymal transition	correlated with tumor stage, and poor prognosis	in RCC and BC
BC 5	MIX E	19	SVNGKVLSK	NOMO1	HLA-A03:01	x	–	x	–	signaling pathways	correlated with tumor stage, associated with aggressive BC	in RCC and BC
		20	RVTYPAKAK	RPN2	HLA-A03:01	x	–	x	p	protein glycosylation	cancer progression	in RCC and BC
		21	ALSDHHIYL	ALDOA	HLA-A02:01	x	–	x	–	glycolysis	tumor progression	in RCC and BC
		22	ALYSGVHKK	SASH1	HLA-A03:01	x	–	x	p	apoptosis	downregulation is associated with poor prognosis in BC	–
		23	TSALPIIQK	PLIN2	HLA-A03:01	x	–	x	sp	lipid metabolism	cancer cell survival and proliferation	in RCC and BC
	MIX F	24	ILDKKVEKV	HSP90AB3P	HLA-A02:01	x	–	x	sp	protein folding and stress response	cancer progression	in RCC
		25	ILMEHIHKL	RPL19	HLA-A02:01	x	–	x	sp	protein synthesis	cancer progression	in RCC
		26	ILDQKINEV	ODC1	HLA-A02:01	x	–	x	–	polyamine synthesis	cancer progression	in RCC and BC
		27	ILTDITKGV	EEF2	HLA-A02:01	x	–	x	sp	protein synthesis	cancer progression	in RCC and BC
		28	NRIKFVIKR	GTF2I	HLA-B27:05	x	–	x	sp	transcription regulation	cancer progression	–

Selected peptides from each patient sample. The best predicted HLA allele per peptide is reported. Each peptide was validated in 2 or 3 HLA-matched healthy donors (HD), as part of one mix, as marked. If the same peptide was detected in our RCC cell line 786-O ligandome (reference in text), it is annotated as “p” (as for peptide); if the peptide itself was not found, but other peptides from the same source protein were present, it is annotated as “sp” (as for source protein). Additional known information about the protein functionality, expression in tumors, and clinical relevance is shown.

### Evaluation of immune response to identified tumor peptides revealed CD8^+^ T cells antigen-specific activation and tumor killing

**Figure 4:**
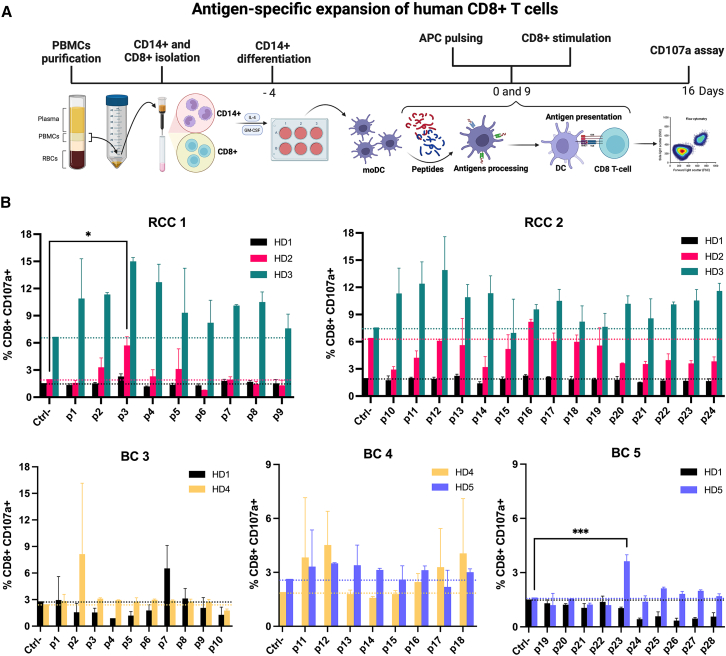
Peptides immunogenicity screening using HLA-matched pulsed CD8^+^ T cells (A) Visual overview of the protocol for antigen-specific stimulation and expansion of human CD8^+^ T cells. PBMCs were purified from healthy donor buffy coats and subsequently CD14^+^ cells and CD8^+^ cells were isolated from PBMCs. CD14^+^ monocytes were differentiated to monocyte-derived DCs (moDCs), seeded together with peptide mixes (5 peptides per mix) and autologous CD8^+^ T cells, in co-culture for 9 days. Subsequently, the same CD8^+^ T cells were stimulated for a second time using freshly thawed CD14^+^ monocytes from the same donor, and co-cultured for 7 days. After an overall 16 days of *in vitro* expansion, CD8^+^ T cells were restimulated with single peptides and their response was analyzed by measuring degranulation through CD107a expression levels by flow cytometry. (B) Flow cytometry analysis measuring surface CD107a marker abundance. The graphs show the responses of CD8^+^ T cells from all healthy donors analyzed, for each patient’s set of peptides. For each replicate (*n* = 2), mean (SD) is shown. Negative control is a CD8^+^ T cell response without any peptide. The dotted line indicates the reference background value per donor. ∗*p* < 0.05, ∗∗*p* < 0.01, and ∗∗∗*p* < 0.001 (one-way ANOVA).

**Figure 5:**
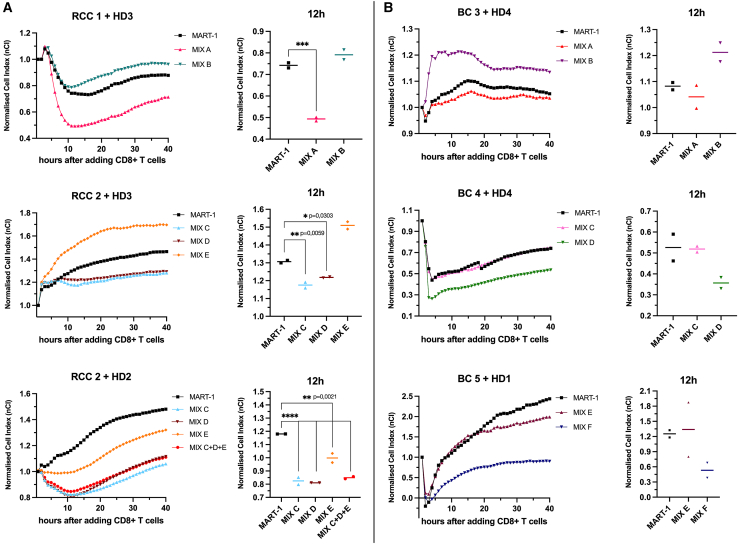
Killing of patient-derived tumor cells by peptide-pulsed CD8^+^ T cells Killing assay using patient-derived tumor cells of RCC (A) and BC (B) and peptide-expanded CD8^+^ T cells as effectors (ratio 1:10). The data shown in the figure are the results of independent experiments performed with representative healthy donors stimulated with different mixes. Mix A and B from RCC1; mix C, D, and E from RCC2; mix A and B from BC3; mix C and D from BC4; and mix E and F from BC5. Mart-1 expanded T cells were used as a negative control for the killing assay. For each replicate (*n* = 2), mean is shown. Normalized cell index (nCI) represents the tumor cell proliferation normalized when the effectors (CD8^+^ T cells) were added to the co-culture. ∗*p* < 0.05, ∗∗*p* < 0.01, and ∗∗∗*p* < 0.001 (one-way ANOVA).

To test the antigens’ capability of eliciting an immune response in an individual, which mainly depends on the presence and frequency of clones within a subject’s T cell repertoire that can recognize the specific epitope, we tested the reactivity of healthy donors’ T cells against the candidate antigens. The immunogenicity can be highly dependent on the HLA allele composition and widely varies between donors. Therefore, we collected healthy donor samples (HD, *n* = 5) with a variety of HLA types (Table S1), matched to the *in silico* predicted ones, to evaluate the antigen-specific T cell response. To this aim, CD8+ T cells were first expanded *in vitro* using a co-culture system with autologous monocyte-derived dendritic cells (moDCs) pulsed with pools of identified tumor-derived peptides (Figure 4A). They were then analyzed for CD107a, a surface marker that increases after antigen stimulation and degranulation, allowing detection of antigen-specific T cells (Figure 4B). Finally, these expanded T cells were tested in patient-derived tumor killing assays (Figure 5).

#### Peptide-stimulated CD8^+^ T cells show antigen-specific CD107a degranulation

Of all the patient-derived epitopes (*n* = 52) that were identified in our cohort from RCC (*n* = 24) and from BC (*n* = 28), several were capable of eliciting peptide-specific CD8^+^T cell responses. Interestingly, peptide-pulsed CD8^+^ T cells exhibited both donor-specific responses as well as more consistent responses across different donors compared to the negative control (CD8^+^ T cells expanded in culture and restimulated with no peptides).

CD8^+^ T cells stimulated with peptide 3 from RCC1 showed a consistent increase in CD107a levels across three healthy donors (HD1, HD2, and HD3), particularly in HD2 (Figure 4B). Responses to peptides 2 and 5 from RCC1 were recurrently higher in HD2 and HD3, while peptides 1, 4, 7, and 8 triggered activity in HD3. For RCC2, peptide 16 elicited increased CD107a expression in both HD2 and HD3, whereas peptides 10–14, 17, and 20, as well as 22–24, showed higher responses in HD3 compared to negative controls. In BC3, peptide 7 induced responses in HD1, while peptide 2 activated cells in HD4. For BC4, peptides 11, 12, 16, and 18 induced strong and recurrent responses in both HD4 and HD5, peptide 17 activated cells in HD4, and peptides 13 and 14 elicited higher reactivity in HD5. Finally, in BC5, peptide 23 from the tumor showed a remarkable increase in CD107a levels in HD5, while peptides 25–27 also induced lower responses in the same donor (Figure 4B).

The well-known highly immunogenic HLA-A2–restricted MART-1 epitope (ELAGIGILTV) was included as a positive control peptide during both the expansion and restimulation phases of T cell culture. Importantly, in most cases, the Mart-1-specific T-cells recognize the positive control peptide (Figures S3B and S4A), which shows that the protocol is able to generate antigen-specific trained T cells. Figures S3C and S4B show dot plots of CD107a-positive CD8^+^ T cells response within the representative RCC or BC peptide-stimulated group compared with the no-peptide restimulated T-cells.

#### Antigen-expanded CD8^+^ T cells demonstrated patient-derived tumor-specific killing

Last, we evaluated the ability of antigen-specific CD8^+^ T cells to recognize and eliminate the tumor by measuring their cytotoxic activity against patient-derived tumor cell cultures (Figure 5) in an *in vitro* killing assay. We measured patient tumor cell lysis after adding CD8^+^ T cells stimulated with tumor peptide mixes or with the Mart-1 peptide and we looked at differences observed between mixes in terms of killing capacity. The peptides were distributed into respective mixes as displayed in Table 1. CD8^+^ T cells cultured with the control peptide Mart-1 also showed background killing against tumor cells (Mart-1 should not be expressed in RCC and BC), whereas stronger cytotoxic responses were observed with peptide mix-expanded T cells compared to Mart-1 expanded T cells, supporting the contribution of tumor peptide-specific recognition.

In the case of RCC (Figure 5A), stimulation of HD3 with RCC mix A (peptides 1–5), which includes peptide 3 previously identified as responsive in the CD107a assay, resulted in T cell recognition and killing of RCC1 tumor cells. Upon stimulation with RCC mix C (peptides 10–14), which includes CD107a-responsive peptides 11–13, and RCC mix D (peptides 15–19), which includes CD107a-responsive peptide 16, specific tumor killing was observed in patient RCC2, both in HD2 and HD3. T cells stimulated with RCC mix E (peptides 20–24) showed noticeable killing of the tumor cells only in HD2 but not in HD3, while RCC mix B (peptides 6–9) did not lead to increased tumor killing.

As for BC (Figure 5B), slightly higher tumor cell killing was observed for both T cells expanded using BC mix D (peptides 15–18) in HD4-containing peptides 16 and 18, which responded to the CD107a assay, and BC mix F (peptides 24–28) in HD1. Conversely, T cells expanded using BC mix A (peptides 1–5), BC mix B (peptides 6–10), BC mix C (peptides 11–14), and BC mix E (peptides 19–23), despite containing peptide 23 from BC5, which appeared to be immunogenic in the CD107a assay, did not exhibit improved killing capacity compared to control-expanded cells.

## Discussion

The selection of relevant tumor antigens and the validation of their therapeutic efficacy represent significant challenges in T cell-based immunotherapy. This study aimed to address these challenges by combining PDCs culture, immunopeptidomics, bioinformatic predictions, and T cell assays to validate the predicted antigens, and assessing CD8^+^ T cell recognition and cytotoxicity toward the same matched patient tumor sample of origin.

Tumor-derived peptides obtained from PeptiCHIP were selected according to our bioinformatic pipeline^19^ and then screened for immunogenicity using HLA-matched healthy donor PBMCs. CD8^+^ T cells were peptide-stimulated and expanded on top of autologous monocytes or moDCs. Subsequently, CD107a degranulation assays confirmed the activation of CD8^+^ T cells in response to specific peptides. Several patient-specific peptides were identified as immunogenic and capable of triggering cytotoxic T cell responses. Co-culture killing assays with tumor PDCs further validated the antitumor immune response of the educated T cells. Notably, some peptides originated from source proteins known to be overexpressed or of diagnostic relevance in RCC and BC (Table 1).

Due to the limited amount of starting material available from patient-derived samples, *ex vivo* expansion was required to enable downstream analyses. For this reason, 2D PDCs were primarily used for their practical advantages, as they allow efficient expansion of tumor cells for functional assays. In the present study, PDCs were therefore not intended to serve as fully patient-matched models but rather as experimentally valid cancer cell models suitable for immunopeptidome analyses and killing assays. Genetic characterization of a representative PDC sample (RCC2) confirmed the retention of key RCC-associated driver mutations, including VHL and PBRM1, supporting their utility as cancer models in this context (Figure S1D). However, comparison of mutation profiles showed that the 3D culture retained a larger fraction of tissue-derived variants than the 2D culture. Consistently, 3D cultures displayed a mutation pattern more closely matching that of the original tumor tissue, whereas 2D cultures showed a less similar profile. Overall, consistent with previous findings,^32^^,^^33^ PDTOs appeared to be more biologically representative of the original tumor, while PDCs remained technically representative but exhibited a lower tumor fraction.

One of the strengths of establishing 3D PDTOs is the preservation of a diverse cellular composition, including tumor cells (which are often epithelial cells in our samples) and stromal fibroblasts, as revealed by immunofluorescence staining (Figures 1B and S1B). Despite some tumors might upregulate it as well, vimentin is a major intermediate filament protein expressed strongly in almost all fibroblasts, which makes vimentin a reliable and robust marker for fibroblasts.^34^ By checking both pan-cytokeratin and cytokeratin 8, we confirmed the presence of epithelial-derived cells in general (pan-CK) and further evaluated CK8 expression, a more specific marker of simple epithelia and many carcinomas, allowing a more refined characterization of the epithelial subtype. The immunofluorescence (IF) imaging confirmed that the cellular composition of the patient's samples closely resembled the *in vivo* tumor microenvironment diversity, reflecting the complexity found within actual tumors, which is typically depleted in culture over time.^4^ This cellular diversity is essential for creating physiologically relevant models to study tumor-immune interactions and evaluate therapeutic responses.

One critical aspect of executing ligandome analysis on samples from patients’ biopsies is that the heterogeneity of the cell populations in the samples makes it difficult to assess whether the retrieved peptides come from tumor cells or other cell types existing within the culture.

The preservation of the immunopeptidome of patient tumors in PDC cultures is something that needs further studies to ensure that they accurately reflect the antigenic landscape of the original tumor, a crucial factor for evaluating T cell-mediated responses to immunotherapies. Although some studies show that patient-derived organoids largely preserve the tumor immunopeptidome and HLA-restricted neoantigen landscape, making them a reliable model for immunopeptidomics and personalized immunotherapy testing.^35^

The HLA expression of our patient's sample (Figure 1C) was usually higher in benign cells compared to their corresponding tumor counterparts (as expected since HLA is often downregulated in tumors), except for RCC2 tumor which exhibited higher HLA levels compared to RCC2 benign, a variation that may align with RCC’s known immunogenic properties.^36^ It is known that HLA expression influences the amount of isolated HLA-I peptides, and consistent with this, flow cytometry analysis revealed low surface levels of pan-HLA in BC5, which could explain the low peptide yield of retrieved peptides from patient BC5.

One significant advancement was the incorporation of a novel ligandome tool developed in our laboratory (PeptiCHIP).^18^ This innovation leverages microfluidics-based strategies for immunopeptidome analysis, offering several advantages, including reduced antibody usage, faster peptide isolation, and minimized tissue requirements, a critical benefit for samples with limited availability as in the case of patients’ tissues.

When interpreting these data, it is important to consider that the HLA ligandome always reflects a dynamic snapshot of antigen presentation at the time of analysis and is strongly influenced by biological factors, such as sample quality, input material, and HLA expression levels, which can vary substantially between samples^37^ (Figure 1C), as well as by technical factors, including mass spectrometry sensitivity and acquisition settings.^38^ Reproducibility is a recognized challenge in immunopeptidomics, particularly for low-input samples, as reduced material inevitably limits peptide yield.^39^ While microfluidic platforms, such as PeptiCHIP, enable analysis of scarce patient-derived material, the resulting ligandome should therefore be interpreted as a partial window of the whole immunopeptidome. Consequently, a partial overlap between biological replicates (separate runs from independently processed samples) is predicted, but complete concordance should not be expected. Nevertheless, even with scarce material, we were still able to identify relevant and immunogenic peptides, demonstrating the practical utility of the method.

Interestingly, several peptides identified from tumor-associated proteins displayed both unique and shared presentation across our RCC and BC immunopeptidomes. Consistent with previous reports, such shared peptides frequently originate from proteins linked to metabolic pathways, immune evasion, or tumor progression.^40^ Given their potential immunogenicity and particular relevance, we prioritized 9-mer peptides, as this canonical length is most efficiently presented by MHC class I molecules, ensuring optimal binding, stability, and T cell recognition.^27^ In contrast, 10-mers, while occasionally presented, are less frequent and generally exhibit lower binding affinity.

Peptide 3 found in RCC1, derived from COL6A3, elicited consistent CD107a degranulation responses across HD1, HD2, and HD3. COL6A3 is a prognostic marker (The Human Protein Atlas) overexpressed in RCC and correlates with poor survival; along with COL5A1 and COL11A1, it is considered a potential diagnostic and therapeutic target in metastatic RCC. Another COL6A3-derived peptide (FRVGNVQEL) was previously found in other ligandomes,^36^ while other COL6A3 peptides (twenty 9-mers and ten 10-mers) were found in our previous RCC cell line 786-O ligandome.^31^

Peptide 4 from RCC1, derived from GAPDH, elicited a response in HD3 but not in HD1 or HD2. GAPDH is increasingly being investigated for its non-glycolytic roles in cancer, with several studies showing its overexpression in RCC and potential tumor-specific immunogenicity.^41^ Antigens derived from GAPDH protein were found in our previously performed ligandome on the RCC cell line 786-O,^31^ underscoring their potential relevance as tumor-associated antigens.

Killing of RCC1 tumor cells by HD3 following stimulation with RCC mix A (which includes both RCC peptides 3 and 4) supports their possible functional relevance.

Peptide 11 identified in RCC2 and included in mix C, stimulated HD3 in the CD107 assay and showed tumor-specific killing by the same donor. Peptide 18, identified in BC4 and included in BC mix D, showed enhanced tumor killing by HD4. These peptides were derived from vimentin, a protein overexpressed in both RCC and BC and associated with tumor grade, stage, and poor prognosis.^42^^,^^43^ Also, some tumors undergoing epithelial to mesenchymal transition might upregulate vimentin. Although thirteen vimentin-derived peptides were found in our previous 786-O ligandome, they were not identical to those detected in our primary BC samples.^31^

Peptide 11 (from DDX3X) and 12 (from CFL2), both found in BC4, induced T cell degranulation in HD4 and HD5. Interestingly, peptide 11 was also found in our 786-O ligandome.^31^ DDX3X is a protein involved in RNA processing and has been linked to tumor progression, while CFL2 plays a role in cytoskeletal dynamics and cancer cell motility.

Peptides 23, 25, 26, and 27 identified in BC5, induced T cell activation in HD5. These peptides were derived from relevant proteins in tumor biology—PLIN2, RPL19, ODC1, and EEF2—which are associated with cancer progression, survival, and proliferation.^44^^,^^45^^,^^46^^,^^47^

Taken together, these data show that at least a subset of the patient tumor cells can be recognized and eliminated by patient-specific antigen-expanded CD8+ T cells, suggesting that some of the identified tumor peptides might be presented on the surface of tumor cells in sufficient amounts for recognition by the specific CD8+T cells.

In this study, the statistical power of the immunogenicity and killing assays is modest, as most experiments were performed with *n* = 2 technical replicates rather than the *n* = 3 typically required for robust statistical analyses. Given practical constraints (including the large number of peptides tested, the limited availability of primary human material, and the high sensitivity of primary T cells to *ex vivo* manipulation), we needed to minimize handling time and experimental complexity. Consequently, these statistical limitations should be considered when interpreting the results.

In addition, our results should be interpreted in the context of the so-called immunopeptidomic “dark matter,” which refers to the large fraction of HLA-presented peptides that are low-abundance, condition-dependent, and not consistently detected across experiments.^48^ Recent evidence increasingly suggests that many HLA-presented peptides can be differentially displayed depending on culture conditions, cellular stress, and the physiological state of the cells, and these factors likely contribute to the large diversity of peptides detected.^49^^,^^50^ Within this framework, the HLA ligandome always represents a dynamic snapshot that is highly influenced by cellular state, including culture conditions, metabolic activity, and cellular stress, all of which can profoundly affect antigen processing and presentation.^51^ Importantly, such variability does not detract from the relevance of these ligands but rather highlights the dynamic nature of antigen presentation and the value of approaches that capture patient- and condition-specific immunopeptidomes.

The relatively small sample size in this study was primarily due to the complexity of obtaining sufficient patient material, including tumor tissue and corresponding benign tissue from the same individual. As these samples consist of primary material, their availability is limited and their capacity for expansion is restricted. Furthermore, inclusion of additional patients would increase HLA diversity, necessitating the identification of more HLA-matched healthy donors for PBMC isolation, adding further logistical challenges. Despite these constraints, the number of samples analyzed is in line with what is currently feasible for patient-specific studies and allowed us to pick up relevant and immunogenic peptides.

Furthermore, we attempted to test patient-derived PBMCs; however, the experiment was unsuccessful due to the low cell viability and the limited quantity available from patient blood samples. Optimization of relevant protocols for cell expansion to overcome these challenges is needed.

In the future, we consider integrating this platform with PeptiCRAd technology,^52^ which enables rapid, versatile, patient-specific loading of tumor antigens found by this pipeline on viral adjuvants. This would provide a highly immunogenic context for antigen delivery and could enhance the platform’s capacity to evaluate personalized vaccine candidates and combination therapies.

In conclusion, this research illustrates a feasible approach for identifying and validating relevant tumor peptides capable of eliciting T cell-mediated immune responses. With further refinement and validation of larger patient cohorts, this platform may support the development and optimization of personalized cancer immunotherapy strategies.

## Materials and methods

### 2D and 3D PDC culture

Human samples were collected under approved permission for the DEDUCER study (HUS/71/2017, HUS/155/2021, HUS/850/2017), through our iCAN-COMPORG subproject.

Fresh tumor and benign material were collected immediately after surgeries, dissociated physically, and treated with the Tissue Dissociation Kit (Miltenyi). Single cells were grown as 3D models on 30% Matrigel (Corning) for approximately 1–3 weeks, in the presence of culture media containing specific growth factors and/or inhibitors required by that tissue, then transferred to plastic or 2% Matrigel for downstream analysis.

RCC samples were cultured in F-medium (3:1 of F-12 nutrient mixture [Ham])-DMEM (Dulbecco’s modified Eagle medium) (Invitrogen), 5% FBS (fetal bovine serum) (Thermo Fisher Scientific), 1% penicillin-streptomycin (Gibco), 0.4 μg/mL hydrocortisone (Sigma), 5 μg/mL insulin (Sigma), 8.4 ng/mL cholera toxin (Sigma), 24 μg/mL adenine (Sigma), 10 ng/mL epidermal growth factor (Corning), and 10 μM ROCK inhibitor (Enzo Life Sciences).

BC samples were cultured in hepatocyte-defined medium (Corning) supplemented with 5% charcoal stripped FBS (Thermo Fisher Scientific), and 1% penicillin-streptomycin (Gibco), 1% GlutaMAX (Gibco), 10 ng/mL epidermal growth factor (Corning), and 10 μM ROCK inhibitor (Enzo Life Sciences).

### HLA typing of PDCs and healthy donor PBMCs

The clinical HLA typing of RCC and BC samples and healthy donor PBMCs was performed by the Finnish Red Cross Blood Service HLA laboratories. Allele determination of three classical HLA-I genes, HLA-A, HLA-B, and HLA-C was performed by the targeted PCR-based next-generation sequence (NGS) technique according to the protocol provided by the manufacturer (NGSgo Workflow, GenDx, Utrecht, Netherlands). The table has been created with BioRender (https://www.biorender.com).

### Microscopy of 3D PDTOs

Cells derived from tumor biopsies were seeded on 30% Matrigel in 96-well plate, and after 2–3 weeks of culture, the PDTOs were imaged using the Invitrogen EVOS M7000 Cell Imaging System (Thermo Fisher Scientific) microscope.

### Immunofluorescence staining of PDCs

PDTOs were disassociated using gentle cell dissociation reagent (STEMCELL Technologies) and 2 × 10^4^ PDCs were plated in 2D on 8-well chamber slides (Nunc; Lab-Tek; II) and allowed to grow for 2 days. Cells were fixed with 4% paraformaldehyde; permeabilized using 0.1% Triton X-100; and stained for CAIX (Novus cat.# NBP1-51691, RRID: AB_11011250), vimentin (2D1) (Novus cat.# NBP1-92687, RRID: AB_11017879), pan-cytokeratin (AE-1/AE-3) (Novus cat.# NBP2-29429, RRID:AB_3068002), cytokeratin 8 (LP3K) (Novus cat.# MAB3165), p53 (Novus cat.# NBP2-59631, RRID: AB_3348568), E-cadherin (ST54-01) (Novus cat.# NBP2-67540), Alexa Fluor 633 Phalloidin (Invitrogen), and DAPI (Sigma), following manufacturer’s instructions. Secondary antibodies were donkey anti-mouse IgG Alexa Fluor 488 (Thermo Fisher Scientific cat.# A-21202, RRID: AB_141607) and donkey anti-rabbit IgG 568 (Thermo Fisher Scientific cat.# A10042, RRID: AB_2534017). Microscopy pictures were taken using the Invitrogen EVOS M7000 Cell Imaging System (Thermo Fisher Scientific).

### Flow cytometry of PDCs

PDTOs were disassociated using a gentle cell dissociation reagent (STEMCELL Technologies), and PDCs were incubated for 30 min at RT with PE anti-human HLA-DR, DP, DQ (BioLegend cat.# 36kkkk1716, RRID: AB_2750318) and APC anti-human CD274 (B7-H1, PD-L1) (BioLegend cat.# 329708, RRID: AB_940360) antibodies. Flow cytometric analysis of RCC and BC samples was performed using a BD Accuri 6 plus (BD Biosciences) and analyzed with FlowJo software (BD Life Sciences RRID: SCR_008520). Data were processed with GraphPad Prism 10.6 (GraphPad Prism Software, USA RRID: SCR_002798).

### WES of patient-derived samples

For library preparation, 50 ng of gDNA was processed according to Twist Library Preparation EF 2.0 with Enzymatic Fragmentation DOC-001239 REV 1.0 and Twist Target Enrichment Protocol DOC-001273 REV 3.0 manual (Twist Bioscience, San Francisco, CA, USA), with the following modifications. The following adapters were used for ligation: PerkinElmer NEXTFLEX unique dual index (UDI), length 10 bp, 5 μL of 10 μM (PerkinElmer, Waltham, MA, USA). Library quantification and quality check were performed using LabChip GX Touch HT High Sensitivity assay (PerkinElmer, Waltham, MA, USA) and Qubit Broad Range DNA Assay (Thermo Fisher Scientific, Waltham, MA, USA). Libraries were pooled to 8-plex reactions according to the concentration (Qubit BR assay). The exome enrichment was performed using Twist Exome 2.0 + Comp.Exome spike-in probes (37,45 Mb). The captured library pools were quantified for sequencing using QuantStudio 5 Collibri Library Quantification Kit (Thermo Fisher Scientific, Waltham, MA, USA) and LabChip GX Touch HT High Sensitivity assay (PerkinElmer, Waltham, MA, USA).

Sequencing was performed with the Illumina NovaSeq 6000 system using S4 flow cell (Illumina, San Diego, CA, USA) and v1.5 chemistry. Read length for the paired-end run was 101 + 10 +10 + 101 bp.

Analysis was performed using Illumina Dragen somatic mode analysis pipeline v4.2, where each tumor sample was compared to a matched normal from peripheral blood.

### Mutations analysis of fresh tumor tissue and derived 3D and 2D cultures

Somatic variant annotation and prioritization were performed using PCGR. Somatic mutations classified functionally and clinically relevant by PCGR were considered in the analysis, including all coding somatic variants identified in oncogenes and tumor suppressor genes.

The library preparation, sequencing, and primary data analysis using DRAGEN RNA pipeline were performed by FIMM Genomics NGS Sequencing unit at the University of Helsinki supported by HiLIFE and Biocenter Finland.

### PeptiCHIP preparation

The PeptiCHIPs were prepared as described previously.^18^ Briefly, the polymer chip featuring free thiol (-SH) functional groups on the surface was precoated with biotin-alkyne under UV. Next, the biotin-functionalized chip was filled with streptavidin (0.1 mg/mL in PBS, Sigma-Aldrich), incubated for 15 min, and rinsed with PBS three times. Finally, biotin anti-human HLA-A, B, and C W6/32 (BioLegend cat.# 311434, RRID:AB_2566253 San Diego, CA), 1.6 mg/mL in PBS, was immobilized on streptavidin-functionalized surfaces by incubating the chip with the antibody and rinsing with PBS.

### HLA class I peptides purification

HLA class I peptides were purified from PDCs of 3 RCC (RCC1 tumor, RCC2 benign, and RCC2 tumor) and 5 BC (BC3 tumor, BC4 benign, BC4 tumor, BC5 benign, and BC5 tumor) samples. Before ligandome analysis, a total of 1 × 10^6^ PDCs per replicate (*n* = 2/sample) were collected by centrifugation at 300 × *g* room temperature (RT), washed in PBS and snap frozen.

For sample preparation, the snap-frozen cell pellets were dissolved at 4°C for 2 h in lysis buffer (150 mM NaCl, 50 mM TRIS-HCl, pH 7.4), protease inhibitors (Thermo Fisher Scientific Pierce, Waltham, MA), and 1% Igepal (Sigma-Aldrich, St. Louis, MO). The lysates were first cleared by low-speed centrifugation for 10 min at 500 × *g*, and then the supernatant was centrifuged for 30 min at 25,000 × *g*. Cleared lysate was loaded into the microfluidic chip, and MHC complex subunits were eluted in 0.1 M acetic acid and desalted using SepPac-C18 cartridges (Waters) according to the protocol previously described by Bassani et al.^53^ Peptides were purified from the MHC class I protein chains by elution with 30% acetonitrile in 0.1% Trifluoroacetic acid (TFA) and dried using vacuum centrifugation (Eppendorf).

### LC-MS/MS analysis of HLA class I peptides

Briefly, each dry sample was dissolved in 10 μL of LCMS solvent A (0.1% formic acid) by dispensing/aspirating 20 times with the micropipette. The nanoElute LC system (Bruker, Bremen, Germany) injected and loaded 10 μL of the sample directly onto the analytical column (Aurora C18, 25 cm long, 75 μm ID, 1.6 μm bead size, Ionopticks, Melbourne, Australia), constantly kept at 50°C by a heating oven (PRSO- V2 oven, Sonation, Biberach, Germany). After washing and loading the sample at a constant pressure of 800 bar, the LC system started a 30-min gradient from 0% to 32% solvent B (acetonitrile and 0.1% formic acid), followed by an increase to 95% B in 5 min, and finally a wash of 10 min at 95% B, all at a flow rate of 300 nL/min. Online liquid chromatography-mass spectrometry (LC-MS) was performed using a Tims TOF Pro mass spectrometer (Bruker) with the CaptiveSpray source, capillary voltage 1,500 V, dry gas flow of 3 L/min, dry gas temperature at 180°C. MS data reduction was enabled. Mass spectra peak detection, maximum intensity was set to 10. Mobilogram peak detection intensity threshold was set to 5,000. Mass range was 300–1,100 *m/z*, and mobility range was 0.6–1.30 V.s/cm_2_. MS/MS was used with three PASEF (parallel accumulation-serial fragmentation) scans (300 ms each) per cycle with a target intensity of 20,000 and intensity threshold of 1,000, considering charge states 0–5. Active exclusion was used with release after 0.4 min, reconsidering precursor if the current intensity is greater than 4-fold the previous intensity, and a mass width of 0.015 *m/z* and a 1/k0 width of 0.015 V.s/cm^2^. Isolation width was defined as 2.00 *m/z* for mass 700 *m/z* and 3.00 *m/z* for mass 800 *m/z*. Collision energy was set as 10.62 eV for 1/k0 0.60 V.s/cm 2 and 51.46 eV for 1/k0 1.30 V.s/cm^2^. Precursor ions were selected using 1 MS repetition and a cycle overlap of 1 with the default intensities/repetitions schedule.

All MS/MS spectra were searched by PEAKS Studio X+ (v10.5 build 16 October 2019) using a target-decoy strategy. The database used was the UniProt Human Reference proteome database (including isoforms, 98,997 entries, downloaded from uniprot.org on 29 January 2021).

A precursor mass tolerance of 20 ppm and a product mass tolerance of 0.02 Da for CID-ITMS2 were used. Enzyme was none, digest mode unspecific, and oxidation of methionine was used as a variable modification, with max three oxidations per peptide. A false discovery rate (FDR) cutoff of 1% was employed at the peptide level. The mass spectrometry proteomics data have been deposited to the ProteomeXchange Consortium via the Pride-asap (RRID: SCR_012052) partner repository with the dataset identifier PXD068567.

### *In silico* analysis of HLA class I peptides

Immunopeptidomics datasets generated from LC-MS/MS analysis were subjected to a series of *in silico* processing and filtering steps to enable peptide annotation, quality control, and prioritization. To address this, eluted peptides were subjected to an initial manual clean-up step based on physical criteria prior to downstream *in silico* analysis. Specifically, peptides were retained only if their calculated molecular mass fell within the range of 850–1,200 Da, which corresponds to the expected mass window of canonical HLA class I ligands (predominantly 8–11 amino acids). This mass range was chosen to exclude both low-mass species likely representing degradation products or contaminants, and higher-mass species that are less efficiently ionized and less reliably detected under the acquisition settings used. In addition, peptides were filtered based on their mass-to-charge (*m/z*) ratio, retaining precursors with *m/z* values between 400 and 615, consistent with the charge states and detection range optimized for HLA class I peptides in the LC-MS/MS setup. This *m/z* window was selected to enrich for peptides that fall within the optimal sensitivity and resolution range of the mass spectrometer, thereby improving confidence in peptide identification and downstream annotation.^37^^,^^54^^,^^55^

Prediction of peptide-HLA binding affinity was performed using NetMHCpan version 4.1. Peptides with a percentile rank below 0.5% were classified as “strong binders,” peptides with ranks between 0.5% and 2% as “weak binders,” and peptides scoring above 2% as “non-binders,” in accordance with standard conventions.

Clustering analysis of peptides into groups based on sequence similarities was performed using the MHCMotifDecon 1.0 tool. The known peptide-binding motifs were obtained from the Motif Viewer section of NetMHCpan 4.1 (DTU Bioinformatics).

Representation of overlap between replicates or benign and tumor samples was performed using Venn diagrams (http://bioinformatics.psb.ugent.be/webtools/Venn/).

Tumor-associated proteins were found using The Human Protein Atlas Database (https://www.proteinatlas.org/).

Predicted immunogenicity was investigated through IEDB (https://www.iedb.org) and our internally developed and publicly available Homology Evaluation of Xenopeptides (HEX) software.^29^ Data were processed with GraphPad Prism 10.6 (GraphPad Prism Software, USA RRID: SCR_002798).

### Peptides

A total of 24 and 28 peptides were selected for RCC and BC samples, respectively, and purchased from Chempeptide Limited (Shanghai, China).

### PBMC purification and CD8^+^/CD14^+^ cell isolation

PBMCs from healthy-donor buffy coats were isolated by the double density isolation method in a 1:1 ratio of PBS-diluted blood and Ficoll (Sigma). PBMCs were separated by centrifugation at 400 × *g* RT for 30 min with no breaks, collected, and centrifuged at 400 × *g* RT for 10 min. Red blood cells were lysed using ACK lysing buffer (Thermo Fisher Scientific) and removed by centrifugation at 400 × *g* for 10 min. Remaining PBMCs were resuspended in PBS-0.5% BSA. Monocytes and T cells were isolated from PBMCs using magnetic beads for CD14 (Miltenyi Biotec) and CD8 (Miltenyi Biotec), respectively, following the manufacturer’s instructions (Miltenyi Biotec).

Cells were cultured in RPMI 1640 (Euroclone) supplemented with 10% FBS (Thermo Fisher Scientific), and 1% penicillin-streptomycin (Thermo Fisher Scientific), 1% GlutaMAX (Thermo Fisher Scientific), 1% NEAA (non-essential amino acids) (Gibco), and 1% sodium pyruvate (Thermo Fisher Scientific).

### Generation of antigen-specific T cells

Similarly as earlier described,^56^^,^^57^ CD14^+^ cells (2 × 10^6^ in 3 mL/well, 6-well plate) were differentiated into moDCs using 800 U/mL IL-4 (Peprotech) and 1,000 U/mL granulocyte-macrophage colony-stimulating factor (GM-CSF; Peprotech) for 4 days. moDC (2 × 10^5^ in 1 mL/well, 24 well) were pulsed with peptide mixes (10 mM of each peptide), Mart-1 (10 mM), or no peptide as control. After 2 h, TNF-α (10 ng/mL, Peprotech) and LPS (10 ng/mL, Sigma) were added for DC maturation. After 4 h, supernatant was removed, and autologous CD8^+^ T cells (2 × 10^6^ in 2 mL/well) were added at a 1:10 ratio. Re-stimulation of the same CD8^+^ T cells was done after 10–12 days using fresh autologous monocytes pulsed with peptide mixes (10 mM of each peptide). Cells were fed with IL-2 (50 U/mL, Proleukin) or IL-15 (10 ng/mL, Peprotech) every 2–3 days. After an overall 16 days of co-culture, CD8^+^ T cells were analyzed by fluorescence-activated cell sorting (FACS). The figure has been created with BioRender (https://www.biorender.com).

### CD107a degranulation assay

Stimulated CD8+ T cells (1 × 10^5^/well) were incubated with autologous monocytes (5 × 10^4^/well) and single peptides (10 μM) or no peptide in the presence of PE-Cy7anti-human CD107a (BioLegend), brefeldin A (eBioscience), and GolgiStop containing monensin (BD Biosciences) in a 96-well plate reaching a final volume of 200 μL. After 5 h incubation, cells were washed in PBS, blocked with PBS-1% FBS for 15 min, washed again, and stained with antibody mix. Samples were incubated with Alexa 488 anti-human CD14 (BioLegend), PerCP anti-human CD8 (BioLegend), and Fixable Live/Dead Viability Dye BV510 (BD Bioscience) for 20 min, then washed, and resuspended in PBS. Flow cytometric analysis was performed using a BD FACS Canto (BD Biosciences) and analyzed with FlowJo software (BD Life Sciences RRID: SCR_008520). Data were processed with GraphPad Prism 10.6 (GraphPad Prism Software, USA RRID: SCR_002798).

### Real-time impedance-based cytotoxicity assay

Tumor killing was measured using impedance-based XCELLigence real-time cell analysis system (ACEA Biosciences). Briefly, 2 × 10^4^ RCC or BC tumor cells were seeded in 100 μL per well in a 16-well E-Plate (ACEA Biosciences). After 24 h, 2 × 10^5^ antigen-pulsed CD8^+^ T cells were added at an effector/target (E/T) ratio of 1:10 in 100 μL per well (reaching a total of 200 μL/well). The cell index (CI) of the target cells was measured every 1 h for the first 24 h and every 15 min after adding the effectors. Data recordings were collected as normalized CI (nCI), being the cell index normalized at the moment the effectors were added. Data were processed with GraphPad Prism 10.6 (GraphPad Prism Software, USA RRID: SCR_002798).

### Statistical analysis

GraphPad Prism 10.6 (GraphPad Prism Software, USA RRID: SCR_002798) was used to perform statistical analysis.

## Data and code availability

The authors confirm that all manuscript data are available in the main text and supplemental information. Raw data of this study are available upon reasonable request from the corresponding author. The mass spectrometry proteomics data have been deposited to the ProteomeXchange Consortium via the PRIDE partner repository under the identifier PXD068567.

## Acknowledgments

We thank all the participants for their support and advice. We appreciate the collaboration and support received from Stephen Full and Adyary Fallarero from 10.13039/100011033Thermo Fisher Scientific. We acknowledge FIMM Genomics NGS Sequencing unit, hosted by the University of Helsinki and supported by 10.13039/100015735HiLIFE and 10.13039/501100013840Biocenter Finland for performing the DNA sequencing (WES) of patient samples to investigate mutation profiling. The clinical HLA typing of patients’ samples (RCC and BC) and healthy donors’ PBMCs was performed by the Finnish Red Cross Blood Service. Finnish Red Cross Blood Service Biobank personnel are acknowledged for organizing research project-specific sample collection in blood donation activities. We thank Humanitas Research Hospital (Milan, Italy) for providing healthy donor buffy coats and facilities (Flow cytometry facility).

This work was supported by the iCAN Digital Precision Cancer Medicine platform, 10.13039/501100002341Academy of Finland (iCAN Flagship), 10.13039/501100004155Magnus Ehrnrooth Foundation, 10.13039/100010116Medicinska Understödsföreningen Liv och Hälsa, The Blood Service Research Fund, The 10.13039/501100003125Finnish Cultural Foundation, 10.13039/501100000781European Research Council under the 10.13039/501100007601Horizon 2020 framework under grant 681219, Finnish Cancer Foundation under grant 4706116, 10.13039/501100004012Jane and Aatos Erkko Foundation under grant 4705796, and Helsinki Institute of Life Science (10.13039/100015735HiLIFE) under grant 797011004. R.M.B. acknowledges funding from Swedish 10.13039/501100002794Cancerfonden (23 2819 Pj 01 H), Swedish Erling Perssons stiftelse (22/9-2020), and 10.13039/501100004359Swedish Research Council (2019-04830).

Author Gabriella Antignani has received a 3-month European Federation of Immunologycal Societis and Immunology letters (EFIS-IL) Fellowship, which allowed mobility to Humanitas Research Institute (Milan, Italy) for the development of crucial technicalities for the project.

The Gene Technology Board has approved working space (GMO permit 15/M15).

We have access to patient samples through our iCAN-COMPORG subproject (clinical collaborator Dr. Antti Rannikko) with approved permissions for the DEDUCER study HUS/71/2017, HUS/155/2021, HUS/850/2017.

## Author contributions

G.A. led the project, wrote the original manuscript draft, and performed the experiments and related data analysis. G.A., M.G., and V.C. designed the study. G.A., M.G., M.Fe., J.C., V.F., and D.C. set up and optimized the methodology. S.F. supervised the use of PeptiCHIP. S.R. helped with figure creation. M.K. and J.S. provided technical support. T.J.L. supported with WES and mutation data analysis. V.M.P. was involved in the sample collection pipeline. M.H. and T.S. fabricated PeptiCHIP. R.M.B. and J.L. performed mass spectrometry. M.R. provided buffy coats and flow cytometry core. S.K. and J.P. contributed to the samples’ HLA typing. M.G., and V.C. supervised the study and were responsible for funding acquisition. All authors interpreted results, read, reviewed, and approved the final manuscript.

## Declaration of interests

V.C. is a co-founder and shareholder at VALO Therapeutics. V.S. is a current employee of AstraZeneca.

## Supplemental information

Document S1. Figures S1–S4 and Table S1

Document S2. Article plus supplemental information
