# Supplementary material for: Patient-derived tumor organoids for personalized cancer immunotherapy: An immunopeptidome-to-validation approach in RCC and BC
Source: Mol Ther Oncol. 2026 May 29;34(3):201247. doi: 10.1016/j.omton.2026.201247 (PMC13312482; doi:10.1016/j.omton.2026.201247)
Supplement: Document S1. Figures S1–S4 and Table S1 [file mmc1.pdf]

OMTON, Volume *34*

Supplemental information

Patient-derived tumor organoids for personalized cancer immunotherapy: An immunopeptidome-to-validation approach in RCC and BC

Gabriella Antignani, Michaela Feodoroff, Jacopo Chiaro, Sara Feola, Salvatore Russo, Firas Hamdan Hissaoui, Manlio Fusciello, Federica D'Alessio, Paolo Bottega, Yvonne Giannoula, Milda Sakalauskaite, Janita Sandberg, Miska Kosonen, Virpi Stigzelius, Tamara J. Luck, Valentina Ferrari, Daniele Ciampi, Markus Haapala, Rui Mamede Branca, Jukka Partanen, Satu Koskela, Maria Rescigno, Tiina Sikanen, Janne Lehtiö, Joseph Ndika, Otto K. Kari, Vilja M. Pietiäinen, Mikaela Grönholm, and Vincenzo Cerullo

Table S1. HLA typing of healthy donors (HD) buffy coats and patient´s sample.
DNA from each healthy donor (n=5) and patient (n=5) was sequenced, results for HLA-A,B,C genes are reported at high resolution (two-field).

DONOR	HLA	ALLELE 1	ALLELE 2
HD 1	HLA-A	02:01	03:01
	HLA-B	50:01	57:01
	HLA-C	06:02	06:02
HD 2	HLA-A	02:01	03:01
	HLA-B	07:02	49:01
	HLA-C	07:01	07:02
HD 3	HLA-A	02:01	03:01
	HLA-B	13:02	58:01
	HLA-C	06:02	07:18
HD 4	HLA-A	03:01	26:01
	HLA-B	18:01	56:01
	HLA-C	01:02	05:01
HD 5	HLA-A	01:01	03:01
	HLA-B	18:01	35:01
	HLA-C	04:01	07:01
**PATIENT**	**HLA**	**ALLELE 1**	**ALLELE 2**
RCC 1	HLA-A	03:01	32:01
	HLA-B	07:02	27:05
	HLA-C	01:02	07:02
RCC 2	HLA-A	02:01	02:01
	HLA-B	35:03	57:01
	HLA-C	04:01	06:02
BC 3	HLA-A	02:01	03:01
	HLA-B	18:01	27:05
	HLA-C	02:02	07:01
BC 4	HLA-A	03:01	03:01
	HLA-B	07:02	07:02
	HLA-C	07:02	07:02
BC 5	HLA-A	02:01	03:01
	HLA-B	07:02	27:05
	HLA-C	02:02	07:02

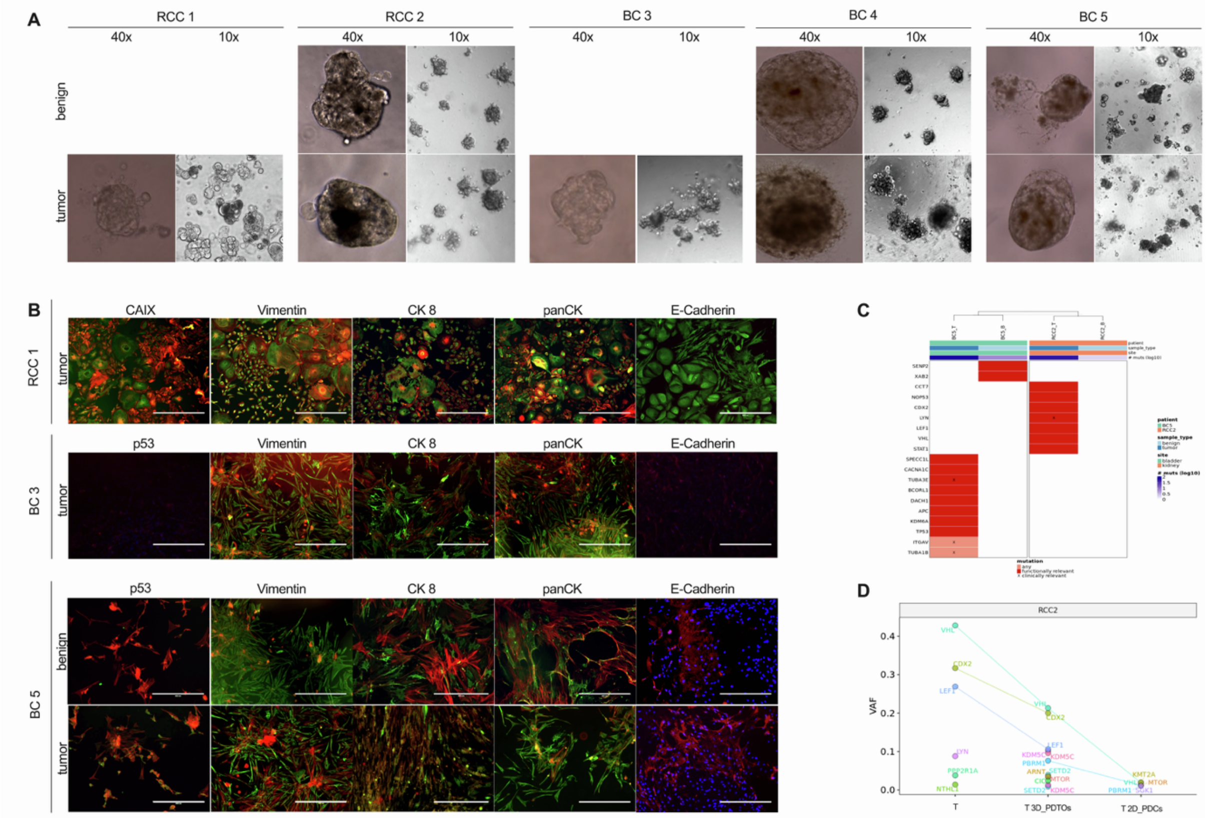

Figure S1. Additional microscope pictures and IF staining for PDTOs characterization: morphology, marker expression and population heterogeneity of patient derived cells.

A) Bright-field microscopy images of patient-derived tumor organoids (PDTOs) from benign and tumor samples of RCC and BC cells cultured in 3D in supportive Matrigel. Pictures were taken an 40x and 10x magnitudes.

B) Immunofluorescence staining using population markers to visualize heterogenous cell cohorts. Benign and tumor patient-derived cells were stained for Vimentin (green), Cytokeratin 8 (green), pan Cytokeratin (green), E-Cadherin (red) which was co-stained with CAIX (green) in RCC. In addition, RCC cells were stained for CAIX (green), while BC cells were assessed for p53 (red) expression. Scale bar 400um.

C) Whole Exome Sequencing (WES) on one representative RCC and one BC shows somatic mutations found in benign and tumor tissue.

D) Comparison of somatic mutations between fresh tumor tissue and derived 3D and 2D cultures. Plots show the variant allele frequency (VAF) of somatic mutations on the y-axis across three sample types on the x-axis: tumor tissue (T), tumor-derived 3D culture (T 3D_PDTOs), and tumor-derived 2D culture (T 2D_PDCs). Each colored dot represents a single unique somatic variant. Variants detected in more than one sample are connected by lines, indicating the presence of the identical mutation across samples (e.g., VHL mutation detected in all three samples). Gene names are indicated next to the corresponding variants. Points labeled with the same gene name but not connected represent distinct mutations within the same gene.

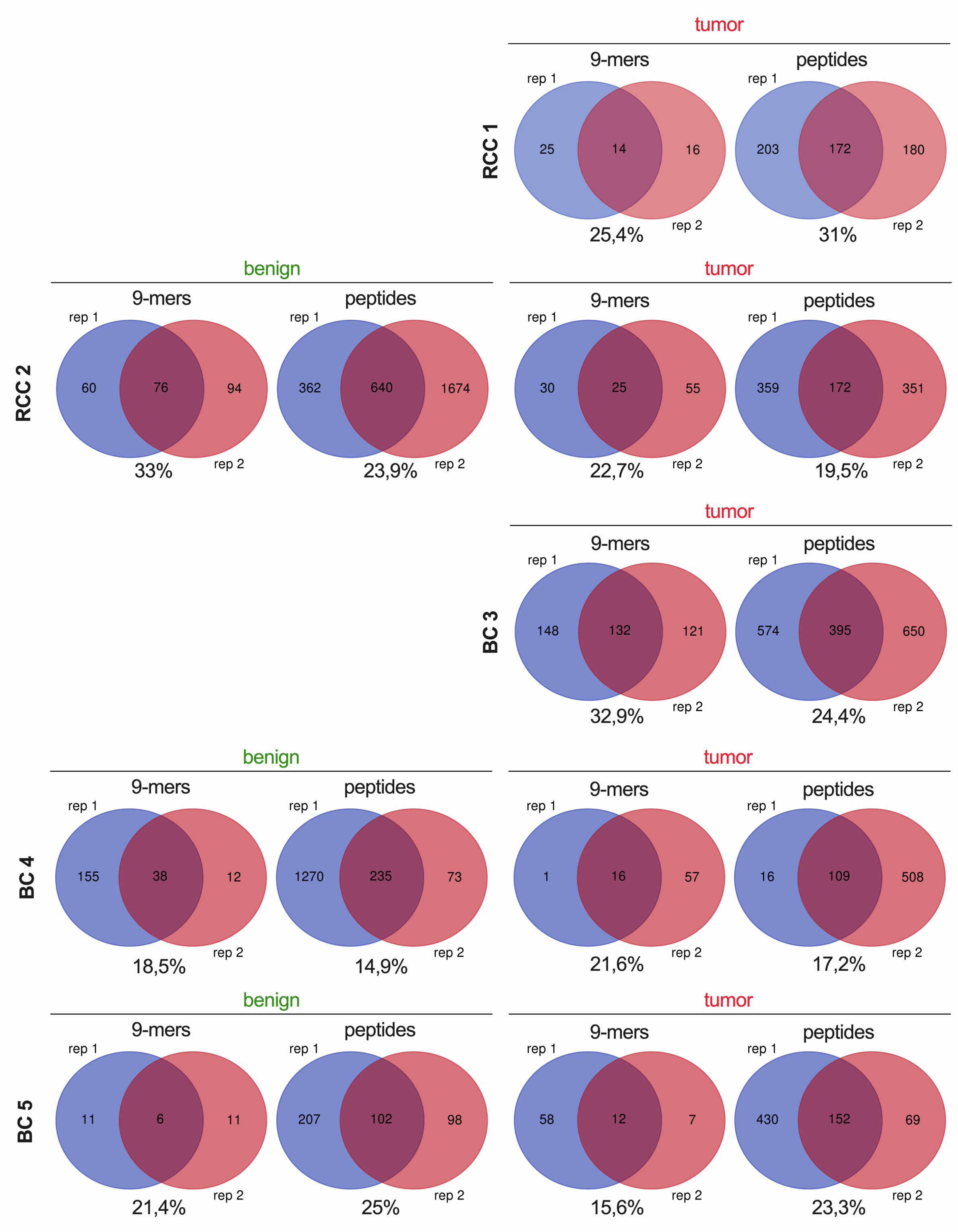

Figure S2. Venn diagrams between replicates (n=2) of each patient´s sample.
Venn diagrams showing overlapping number and percentage after intersecting unique peptides or 9-mers from replicates of the same sample.

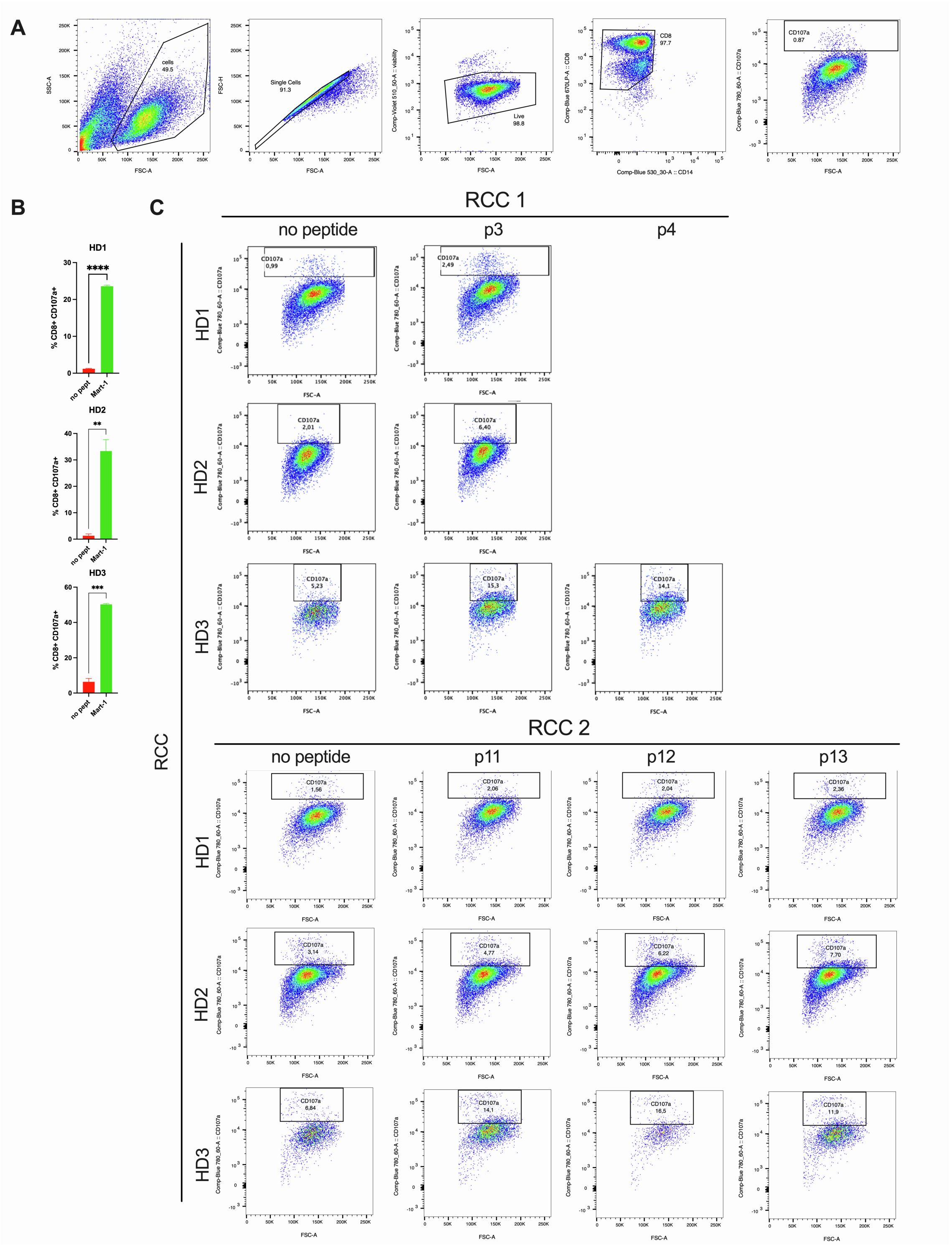

Figure S3. Positive control (Mart-1) CD107a response and Dot plots for the best responding RCC peptides and gating strategy.

Cells were first selected based on forward scatter (FSC) and side scatter (SSC) to exclude debris. Singlets were then identified using a SSC-H versus SSC-A plot. Live cells were gated based on viability dye exclusion. CD8^{+} lymphocytes were defined by CD8 expression while excluding CD14^{+} monocytes. Finally, degranulating cells were identified as CD107a^{+} cells using CD107a expression versus SSC-A (**A**). Flow cytometry analysis of stimulated and expanded CD8+ T cells showing expression levels of CD107a molecules on the CD8+ T cells surface when restimulated with positive control peptide (Mart-1) for each healthy donor (**B**). Dot plots showing CD107a positive CD8+ T cells response within representative RCC (**C**) peptide-stimulated group compared with the no-peptide restimulated T-cells.

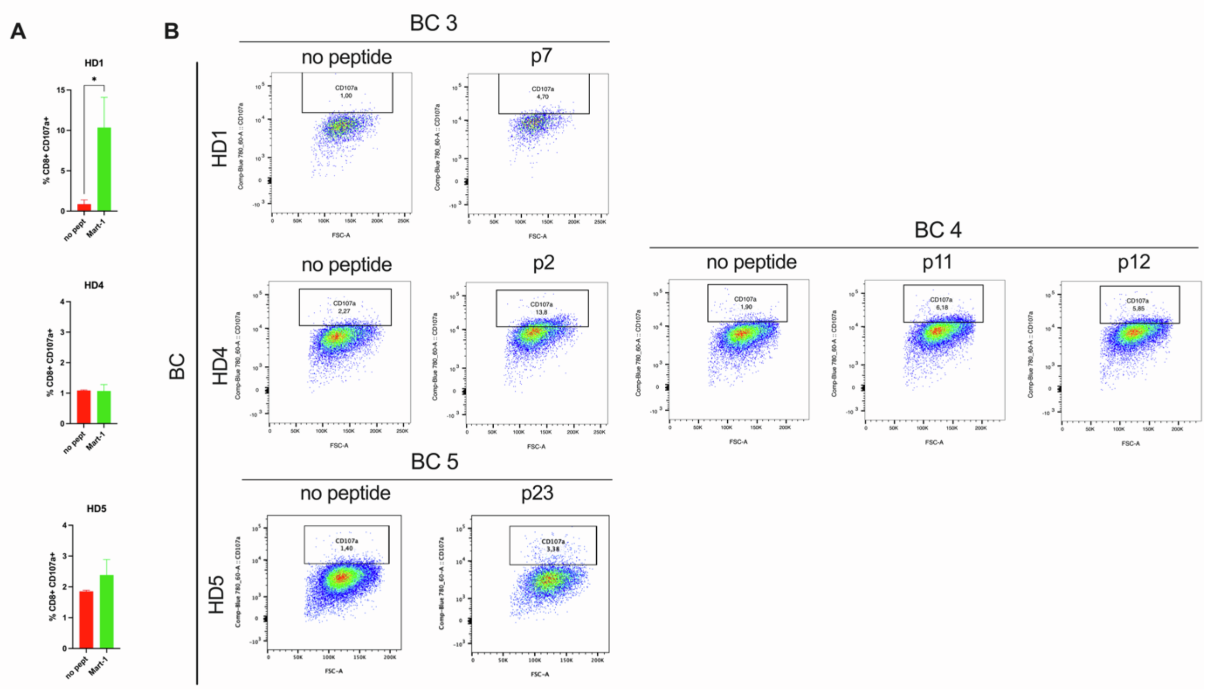

Figure S4. Positive control (Mart-1) CD107a response and Dot plots for the best responding BC peptides.

Flow cytometry analysis of stimulated and expanded CD8+ T cells showing expression levels of CD107a molecules on the CD8+ T cells surface when restimulated with positive control peptide (Mart-1) for each healthy donor (**A**). Dot plots showing CD107a positive CD8+ T cells response within representative BC (**B**) peptide-stimulated group compared with the no-peptide restimulated T-cells.
